# Parental legacy and regulatory novelty in *Brachypodium* diurnal transcriptomes accompanying their polyploidy

**DOI:** 10.1093/nargab/lqaa067

**Published:** 2020-09-22

**Authors:** Komaki Inoue, Kotaro Takahagi, Yusuke Kouzai, Satoru Koda, Minami Shimizu, Yukiko Uehara-Yamaguchi, Risa Nakayama, Toshie Kita, Yoshihiko Onda, Toshihisa Nomura, Hidetoshi Matsui, Kiyotaka Nagaki, Ryuei Nishii, Keiichi Mochida

**Affiliations:** RIKEN Center for Sustainable Resource Science, Tsurumi-ku, Yokohama, 230-0045, Japan; RIKEN Center for Sustainable Resource Science, Tsurumi-ku, Yokohama, 230-0045, Japan; Kihara Institute for Biological Research, Yokohama City University, Totsuka-ku, Yokohama, 244-0813, Japan; Graduate School of Nanobioscience, Yokohama City University, Kanazawa-ku, Yokohama, 236-0027, Japan; RIKEN Center for Sustainable Resource Science, Tsurumi-ku, Yokohama, 230-0045, Japan; Graduate School of Mathematics, Kyushu University, Fukuoka, 819-0395, Japan; RIKEN Center for Sustainable Resource Science, Tsurumi-ku, Yokohama, 230-0045, Japan; RIKEN Center for Sustainable Resource Science, Tsurumi-ku, Yokohama, 230-0045, Japan; RIKEN Center for Sustainable Resource Science, Tsurumi-ku, Yokohama, 230-0045, Japan; RIKEN Center for Sustainable Resource Science, Tsurumi-ku, Yokohama, 230-0045, Japan; RIKEN Center for Sustainable Resource Science, Tsurumi-ku, Yokohama, 230-0045, Japan; RIKEN Center for Sustainable Resource Science, Tsurumi-ku, Yokohama, 230-0045, Japan; RIKEN Baton Zone Program, Tsurumi-ku, Yokohama, 230-0045, Japan; Faculty of Data Science, Shiga University, Hikone, 522-8522, Japan; Institute of Plant Science and Resources, Okayama University,710-0046, Kurashiki, Japan; School of Information and Data Science, Nagasaki University, Nagasaki, 852-8131, Japan; RIKEN Center for Sustainable Resource Science, Tsurumi-ku, Yokohama, 230-0045, Japan; Kihara Institute for Biological Research, Yokohama City University, Totsuka-ku, Yokohama, 244-0813, Japan; Graduate School of Nanobioscience, Yokohama City University, Kanazawa-ku, Yokohama, 236-0027, Japan; RIKEN Baton Zone Program, Tsurumi-ku, Yokohama, 230-0045, Japan; Institute of Plant Science and Resources, Okayama University,710-0046, Kurashiki, Japan

## Abstract

Polyploidy is a widespread phenomenon in eukaryotes that can lead to phenotypic novelty and has important implications for evolution and diversification. The modification of phenotypes in polyploids relative to their diploid progenitors may be associated with altered gene expression. However, it is largely unknown how interactions between duplicated genes affect their diurnal expression in allopolyploid species. In this study, we explored parental legacy and hybrid novelty in the transcriptomes of an allopolyploid species and its diploid progenitors. We compared the diurnal transcriptomes of representative *Brachypodium* cytotypes, including the allotetraploid *Brachypodium hybridum* and its diploid progenitors *Brachypodium distachyon* and *Brachypodium stacei*. We also artificially induced an autotetraploid *B. distachyon*. We identified patterns of homoeolog expression bias (HEB) across *Brachypodium* cytotypes and time-dependent gain and loss of HEB in *B. hybridum*. Furthermore, we established that many genes with diurnal expression experienced HEB, while their expression patterns and peak times were correlated between homoeologs in *B. hybridum* relative to *B. distachyon* and *B. stacei*, suggesting diurnal synchronization of homoeolog expression in *B. hybridum*. Our findings provide insight into the parental legacy and hybrid novelty associated with polyploidy in *Brachypodium*, and highlight the evolutionary consequences of diurnal transcriptional regulation that accompanied allopolyploidy.

## INTRODUCTION

Polyploidy is a widespread phenomenon across eukaryotes that has important evolutionary consequences (1). Whole-genome duplication (WGD) events resulting from chromosome doubling are common, especially in plants (2–4). Previous studies on chromosome numbers from various plant species suggested that 57% (5) to 70% (6,7) of all flowering plants are polyploids (8). Recent genomics-based studies also suggested that all angiosperm species underwent one or more WGD events during their evolutionary history and are thus paleopolyploids (9).

There are two main classes of polyploidy, defined according to the source of the duplicated genome: autopolyploidy, which arises from chromosome doubling within a single species, and allopolyploidy, which arises from interspecies hybridization, followed by chromosome doubling to allow subsequent balanced meiotic divisions (10,11). Allopolyploids that originated from hybridization between diverged species may benefit immediately from genetic and functional innovations, and they often show better growth and adaptability to environments than their ancestors. In addition, allopolyploids behave genetically as fixed heterozygotes, which may result in heterosis and a selective advantage relative to their progenitors. Because genetic redundancy potentially facilitates adaptive divergence of duplicated genes, autopolyploids may also show an increased adaptive potential compared with their diploid ancestors, leading to successful range expansion and radiation (12,13).

Phenotypic modifications in polyploids may be due to altered levels of gene expression compared with their progenitors. Transcriptome changes accompanying polyploidy have been examined in various allopolyploids (14–17), and these studies have demonstrated multiple phenomena related to gene expression of homoeologs that are derived from each parental genome and brought together by allopolyploidy. The expression pattern of homoeologs in allopolyploids can be additive (i.e. each contributing half of the observed expression levels), which can be considered as a simple case of ‘parental legacy.’ However, expression of homoeologs also can be non-additive, leading to ‘hybrid novelty,’ compared with their expression patterns in progenitors. Non-additive expression levels of homoeologs may be caused by expression level dominance (ELD), such that the total expression level of homoeologs is similar to that of one of its parental species. In addition, transgressive expression may occur if expression of the homeologs is outside the range of either parent (18,19). The relative expression level of homoeologs in allopolyploids can also be classified as ‘even’ or ‘uneven,’ depending on the relative contribution of each homoeolog to total gene expression (also known as homoeolog expression bias, HEB) (20–22). HEB can be a legacy derived from progenitors with similar differences in expression levels (bias retention) or can result from altered expression between progenitors (bias gain, bias loss or bias switch) that may lead to hybrid novelty in gene expression accompanying allopolyploidy. HEB may also vary between tissues (23), developmental stages (24,25) and environmental conditions (26). Compared to the multiple studies based on comparative transcriptome analyses between allopolyploids and related diploids, examples of comparative studies with autopolyploids are limited. However, the few studies addressing direct comparisons between related auto- and allopolyploid species have lent support to the hypothesis that autopolyploids present weaker alterations in their transcriptome than allopolyploids due to the absence of genomic hybridization (27).

The study of parental legacies and hybrid novelties in allopolyploid gene expression allows the dissection of the fate of duplicated genes and their associated gene expression profiles. As noted in the review by Buggs *et al.* (28), the parental diploid progenitors of any allopolyploid no longer exist, forcing us to use closely related diploid lineages (diploid sister genomes) as substitutes for the original parental diploid progenitors that gave rise to each subgenome of an allopolyploid. Therefore, any alteration in gene expression between an allopolyploid and its closely related diploid lineages may stem not only from genomic hybridization but also from divergence between the allopolyploid subgenomes and the diploid sister genomes over the course of their independent evolutionary trajectories since the emergence of the allopolyploid (28) . Moreover, these genomic hybridization effects may combinatorially influence gene expression levels in allopolyploids as a direct consequence of the functional divergence of both *cis*-regulatory and *trans*-acting factors (29). Additionally, the dosage effect of duplicated genes resulting from chromosome doubling may also affect gene expression in both auto- and allopolyploids (30).

*Brachypodium* has recently gained attention as a tractable model genus to explore the evolutionary consequences accompanying allopolyploidy. Indeed, three representative cytotypes have emerged for the exploration of parental legacy and hybrid novelty in gene expression patterns following allopolyploidization (31). The allopolyploid *B. hybridum* (2n = 4x = 30, *x* = 10 + 5, 509 Mbp) is derived from a natural hybridization event between *B. distachyon* (2n = 2x = 10, 272 Mbp) and *Brachypodium stacei* (2n = 2x = 20, 234 Mbp), which occurred roughly ∼1 Mya (31,32). Ecological studies illustrated that the two diploid progenitors occupy different habitats (*B. distachyon*: higher, cooler and wetter places, north of 33°; *B. stacei*: lower, warmer and drier places, south of 40° 30′), whereas the allopolyploid has colonized intermediate habitats across these latitudinal boundaries (33). We exploited the fully annotated reference genome sequences of *B. distachyon*, as well as deep sequencing of transcriptome (RNA-seq) datasets of *Brachypodium hybridum* carrying the subgenomes inherited from *B. distachyon* (Bd-subgenome) and *B. stacei* (Bs-subgenome), to quantify homoeolog-specific gene expression in *B. hybridum*. We then examined additivity and non-additivity in transcript levels, and the pattern of HEB in *B. hybridum* (34). We demonstrated that early transcriptional responses of homoeologs likely to be a legacy of gene expression changes from the *B. stacei* subgenome may have contributed to heat stress tolerance in *B. hybridum*, highlighting the role of homoeolog-specific transcriptional regulation in acclimation to the environment (34). These ecological and genomic features of the three *Brachypodium* cytotypes may facilitate a comprehensive analysis of their HEB, providing insight into the modulation of their gene expression patterns during the evolutionary trajectories of the respective cytotypes.

Over the past decade, the circadian clock has gained recognition as a major regulator of various physiological alterations underlying vigor traits observed in plant hybrids and allopolyploids (35–38). Genetic distance between geographically diverse Arabidopsis (*Arabidopsis thaliana*) accessions corresponded with natural variation of circadian rhythms and the expression of clock-controlled stress-responsive genes in intraspecific hybrids (39). Transcriptome profiling of hexaploid wheat (*Triticum aestivum*) provided evidence of ELD in genes involved in photoperiodic flowering, such as *LATE ELONGATED HYPOTOCYL* (*LHY*) and *CONSTANS* (*CO*), which suggests a potential contribution to flowering-related adaptations (17). Since the plant circadian clock influences various traits related to environmental fitness such as stress tolerance, defense and latitudinal adaptation (40), circadian plasticity may facilitate local adaptation and ecological speciation (41). However, HEB and its evolutionary consequences following polyploidization in the context of diurnal rhythms has not been fully described in plant allopolyploids or their diploid progenitors.

To gain a deeper understanding of the expression fates of duplicated genes in plant polyploids, we present here the transcriptomic landscape of parental legacy and hybrid novelty along with the rhythmic diurnal expression profile associated with polyploidy in *Brachypodium*. We first aimed to assess the alterations of the transcriptome in auto- and allopolyploid species through profiling and direct comparisons of diurnal transcriptomes from *B. distachyon*, *B. stacei*, *B. hybridum* and an artificially induced autotetraploid *B. distachyon*. We identified commonly regulated genes in the diurnal transcriptome of *B. hybridum* and the *B. distachyon* autotetraploid, and estimated the influence of chromosome doubling on gene expression that accompanies polyploidy in *Brachypodium*. We then aimed to examine parental legacy detected in *B. hybridum* through a comparative analysis of HEB patterns between the subgenomes in *B. hybridum* and the diploid sister genomes of *B. distachyon* and *B. stacei*. We quantified the conserved HEB and balanced expression level of homoeologs in *B. hybridum*. Finally, we aimed to examine HEB in diurnally expressed genes and putative homologs of clock-related genes, which may be rhythmically regulated by *trans*-acting factors from both homoeologs. We observed a tighter correlation between the diurnal phase of homoeologs in polyploids when compared to their diploid progenitors, suggesting an averaging effect of subgenome-specific *trans* effects as a mode of hybrid regulatory novelty by compensatory *cis*- and *trans*-effects in allopolyploids.

## MATERIALS AND METHODS

### Plant materials

The allotetraploid *B. hybridum* Bd14–1 and its diploid progenitor species, *B. distachyon* Bd21 and *B. stacei* ABR114, were obtained from the National Plant Germplasm System of the United States Department of Agriculture–Agricultural Research Service (David F. Garvin; Plant Science Research Unit, University of Minnesota, USA) and Pilar Catalán (Department of Agriculture and Environment Science, High Polytechnic School of Huesca, University of Zaragoza, Spain). We induced the generation of a *B. distachyon* Bd21 autotetraploid by treating Bd21 seedlings with a colchicine solution (0.05% colchicine, 2% Dimethyl sulfoxide (DMSO) and two drops Tween-20) for 24 h before transplanting them to soil (42).

### B. *stacei* virtual genome

To analyze homoeolog-specific gene expression in *B. hybridum*, we generated a virtual *B. stacei* genome by replacing nucleotides in the *B. distachyon* genome with the corresponding single nucleotide polymorphisms (SNPs) from the *B. stacei* genome, as documented by Takahagi *et al.* (34). Briefly, we mapped whole-genome sequencing data from *B. hybridum* and *B. stacei* (available at the DNA Data Bank of Japan under accession number DRA005717) to the reference *B. distachyon* Bd21 genome, and identified SNPs in homoeologs between these *Brachypodium* species. We selected SNPs that discriminated between homoeologs based on the following criteria: homogenic across all *B. stacei* reads and heterogenic in *B. hybridum* reads. We then replaced nucleotide sequences at homoeologous SNP positions in the reference *B. distachyon* Bd21 genome with those from *B. stacei* to generate a virtual (*in silico*) collection of homoeolog sequences from *B. stacei*. The virtual *B. stacei* genome sequence dataset is available at the GigaDB website (40).

### Flow cytometry, chromosome observations and whole-genome resequencing

We filtered leaf cells, stained with 4′,6-Diamidino-2-phenylindole (DAPI, Thermo Fisher Scientific, Waltham, MA, USA), through a 30 μm nylon mesh and analyzed their DNA content by flow cytometry (CyFlow space, Sysmex Corp., Kobe, Japan) to identify plants with duplicated genomes. We obtained self-pollinated seeds from colchicine-treated *B. distachyon* Bd21.

To observe somatic chromosomes, we germinated self-pollinated seeds obtained from colchicine-treated *B. distachyon* Bd21 on moist filter paper at 22°C. We immersed germinated roots (∼3 cm long) in ice-cold water for 24 h, followed by fixation in 3:1 methanol:acetic acid. After two 10 min washes in water, we excised root tips from the roots and digested the root tips with a mixture of 1% (w/v) cellulase Onozuka RS (Yakult Pharmaceutical Industry, Tokyo, Japan) and 0.5% (w/v) pectolyase Y-23 (Seishin Pharmaceuticals, Tokyo, Japan) for 2 h at 37°C. After another two 10-min washes in water, we fixed root tips for 10 min and then resuspended them in fixative solution. We then spread the resuspended cells onto glass slides by a flame-dry technique, followed by mounting in SlowFade Diamond containing 1 μg/ml DAPI. We captured stained chromosomes using a microscope with a chilled charge-coupled device camera (AxioCam HR, Carl Zeiss, Oberkochen, Germany).

To examine sequence polymorphisms in the induced *B. distachyon* Bd21 autotetraploid, we extracted genomic DNA from leaves using the DNeasy Plant Mini Kit (Qiagen, Tokyo, Japan). We assessed the quality of the extracted DNA on an Agilent 2100 Bioanalyzer (Agilent Technologies, Santa Clara, CA, USA), and prepared a sequencing library using the TruSeq Nano DNA Library prep kit (Illumina Inc., San Diego, CA, USA). We then sequenced this library on an Illumina HiSeq 2500 platform using the TruSeq Rapid SBS Kit (Illumina) and the paired-end sequencing method to obtain 2 × 100 bp sequences. We mapped quality-checked reads to the reference *B. distachyon* Bd21 genome, retrieved from Phytozome (Bdistachyon_314_v3.0, https://phytozome.jgi.doe.gov/pz/portal.html) (43), using TMAP with the mapall, stage1 and map4 commands. We used mapping results to explore sequence polymorphisms between the induced autotetraploid in Bd21 and the Bd21 reference genome.

### Transcriptome analysis

#### Experimental growth conditions

We incubated dry seeds of *B. hybridum* Bd14–1, *B. distachyon* Bd21, *B. stacei* ABR114 and 4×Bd21 on wet filter paper in a Petri dish at 4°C in darkness for 6–7 days to synchronize germination. We then transplanted germinated seeds to pots filled with autoclaved Pro-Mix^®^ BX Mycorrhizae™ (Premier Tech, Quebec, Canada) potting mix and allowed them to grow in a growth chamber at 22°C under a 20 h daylength (100 μmol/m^2^/s). Plants were watered with Professional Hyponex 10–30-20 (Hyponex Japan, Osaka, Japan) fertilizer, diluted 1: 5000 every 3–4 days.

#### RNA sequencing and analysis

For comparative diurnal transcriptome analysis regarding gene expression differences between the *Brachypodium* species, we collected leaf tissue from *Brachypodium* plants every 4 h over 24 h. We entrained seedlings for 3 days in 20 h light/4 h dark cycles (light: 4:00–24:00, dark: 24:00–4:00), a long day condition used by others for *B. distachyon* (44–46), after being transplanted to pots. We performed RNA-seq analysis with three biological replicates per time point and genotype, as described previously (47). We extracted total RNA using the RNeasy Plant Mini Kit (Qiagen) according to the manufacturer's instructions and assessed RNA quality and concentration using an Agilent 2100 Bioanalyzer (Agilent Technologies). We constructed stranded libraries using the TruSeq Stranded mRNA Sample Preparation Kit (Illumina) according to the manufacturer's instructions and assessed them using an Agilent 2100 Bioanalyzer (Agilent Technologies). We generated library clonal clusters using a cBot with the TruSeq PE Cluster Kit (Illumina) and sequenced all libraries on a HiSeq 4000 platform (Illumina) using the TruSeq SBS Kit (Illumina) and the paired-end sequencing method to obtain 2 × 100 bp sequences. Quality checks of RNA-seq reads, mapping reads to the reference genomes, homoeologous reads sorting, and read counting were according to the methods described in (34) with small modifications. Briefly, we trimmed RNA-seq reads using Trimmomatic (v0.32) (48) with the LEADING:20, TRAILING:20, SLIDINGWINDOW:4:15 and MINLEN:36 parameters. We then mapped trimmed reads from *B. distachyon* to the Bd21 genome, while we mapped *B. stacei* reads to our virtual *B. stacei* genome (34). We mapped reads from *B. hybridum* to both genomes using TMAP (https://github.com/iontorrent/TMAP) with the mapall, stage1 and map4 commands. We obtained read counts using featureCounts (v.1.4.6) (43).

#### Homoeolog-specific RNA-seq read sorting

We mapped RNA-seq reads from *B. hybridum* to the *B. distachyon* Bd21 reference genome and *B. stacei* virtual genome. To compute the expression levels of the homoeologs in *B. hybridum*, we divided the origin of RNA-seq reads into *Bd*- and *Bs*-subgenome reads based on the number of mismatches between the read pairs (fragments) and the *B. distachyon* and *B. stacei* genomes, respectively, using a custom Perl script (the script code is available https://github.com/BioproductivityInformaticsResearchTeam/Inoue_and_Takahagi_etal/tree/master/Homoeolog_sorting).

#### Gene expression analysis

Genes showing reads per million mapped reads (RPM) ≥ 1 in all three biological replicates from at least one sampling time were defined as expressed; we used those genes to look for differentially expressed genes (DEGs). We examined DEGs between the polyploid and diploid *Brachypodium* species, and homoeologs using the DESeq2 package (v. 1.18.1) (49) in R (v. 3.4.3). We performed a Wald test based on read count data. We calculated the false discovery rate (FDR) for each comparison by adjusting the *P*-value using the Benjamini–Hochberg procedure. Genes with FDR < 0.01 were defined as differentially expressed.

#### Gene ontology enrichment analysis

We obtained gene ontology (GO) annotations for *B. distachyon* genes as previously described (34). We identified enriched GO terms for selected genes using the GOStats library in R (50) with the hyper geometric test (*P*-value < 0.0001).

### Rhythmically expressed genes

We used the wavelet transformation technique implemented into the WaveletComp package in R (https://cran.r-project.org/web/packages/WaveletComp/WaveletComp.pdf) to identify rhythmically expressed genes. We computed *P*-values for significance of periodic power spectra for the diurnal transcriptome datasets from *B. distachyon*, *B. stacei* and *B. hybridum*; we selected genes with highly significant power (*P* < 0.05) of the 24 h cycle as candidates for diurnally expressed genes across the diurnal transcriptome. In addition, using randomization selection between the three biological replicates of the diurnal transcriptome datasets (enabling six permutations), genes with significant periodicity for all permutations were considered rhythmic in the diurnal transcriptome. We compiled genes from *B. distachyon*, *B. stacei* and *B. hybridum* (*P* < 0.05 in all six permutation tests in at least one species), and estimated their peak time of gene expression in *B. distachyon*, *B. stacei*, *B. hybridum*, *Bd*-subgenome and *Bs*-subgenome based on the diurnal transcriptome datasets with the WaveletComp package.

## RESULTS

### *Brachypodium* autopolyploid

**Figure 1.**
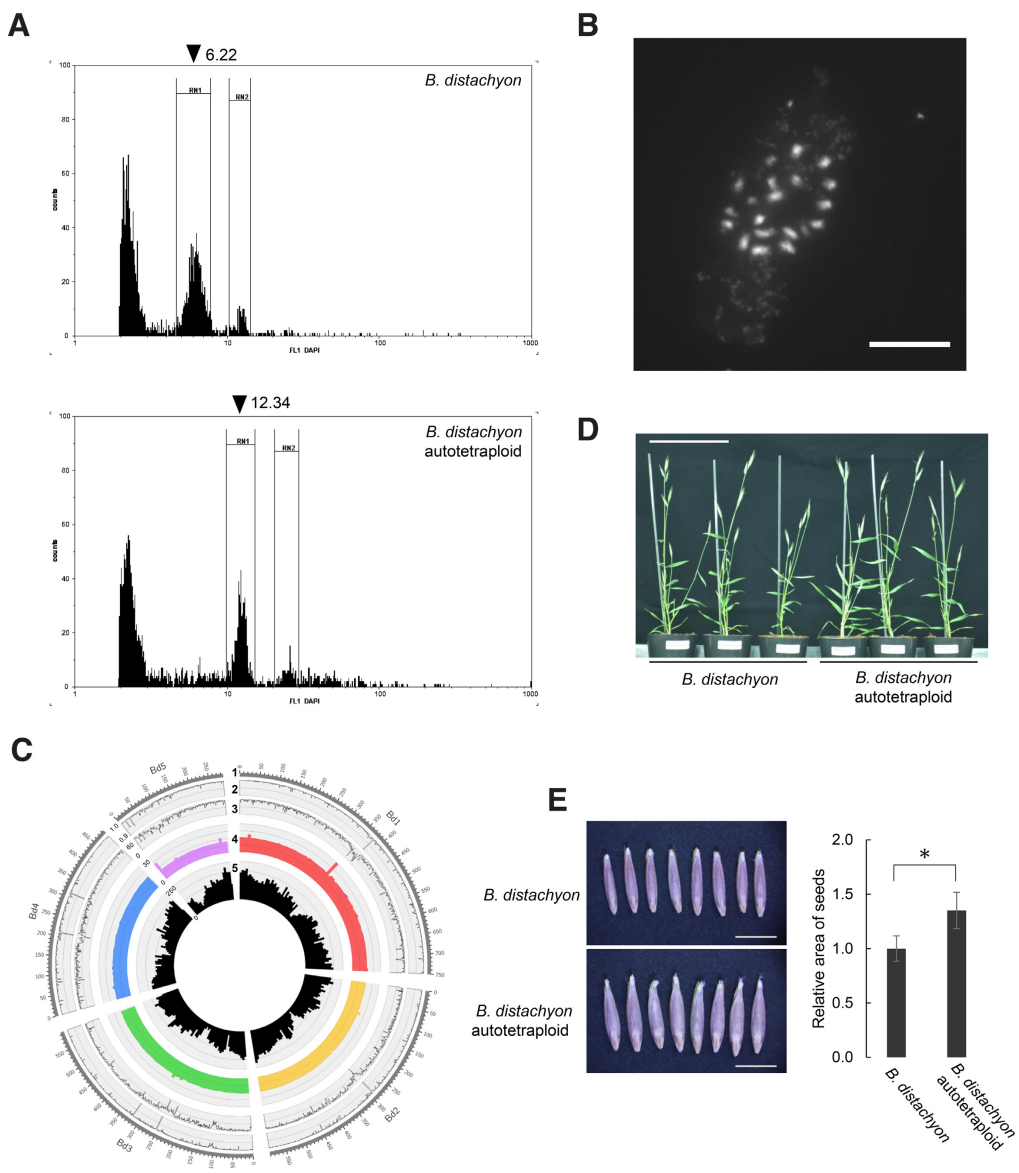
Genome size, chromosome number, genomic sequence, and morphology of the artificially induced *Brachypodium distachyon* tetraploid. (**A**) Flow cytometry profiles for the diploid *B. distachyon* Bd21 and the colchicine-induced autotetraploid Bd21 strain. (**B**) Fluorescence-microscopy of the somatic chromosomes of autotetraploid Bd21 strain. Scale bar = 10 μm. (**C**) Circular representation of whole-genome resequencing results of autotetraploid Bd21 compared to the Bd21 reference genome. 1: nucleotide position on the reference Bd21 genome, 2: percent of nucleotides identical to the reference sequence per 100-kbp window, 3: mean value of root mean square of mapping quality per 100 kbp, 4: mean coverage of mapped reads per 1 Mbp, 5: mean gene density per 1 Mbp. (**D**) Representative diploid Bd21 and autotetraploid Bd21 plants. Scale bar = 10 cm. (**E**) Grain morphology in Bd21 and autotetraploid Bd21. Scale bar = 5 mm. Bar graph represents relative area of seed grains of autotetraploid Bd21 (*n* = 67) compared with those of Bd21 (*n* = 86). *P* < 0.001 is represented by ‘*’ based on Student's *t*-test. Error bars represent standard deviation.

To investigate ploidy-dependent gene expression changes, we induced an autotetraploid *B. distachyon* Bd21 through colchicine treatment. We identified a tetraploid Bd21 individual from self-fertilized progeny using flow cytometry and compared their profile to the diploid Bd21 for genome size estimation. We estimated the genome size of the tetraploid to be 540 Mbp, compared to ∼270 Mbp for the diploid Bd21 (Figure 1A). By observing somatic metaphase chromosomes, we confirmed a doubling of chromosome number in tetraploid Bd21 plants (Figure 1B) compared with diploid Bd21 (51). We also carried out whole-genome resequencing of the tetraploid Bd21 line and confirmed that no significant polymorphisms existed between the tetraploid Bd21 and the reference genome, except for genomic regions showing low mapping scores, likely due to multi-mapping of re-sequenced reads (Figure 1C). These results indicated that the tetraploid *B. distachyon* Bd21 had a fully duplicated Bd21 genome. By comparing growth characteristics of tetraploid and diploid Bd21 plants (Figure 1D), we observed that the grain size of tetraploid Bd21 was larger than in the diploid Bd21 (Figure 1E). Together with the three cytotypes *B. distachyon*, *B. stacei* and *B. hybridum*, this new artificially induced and genome-sequenced *B. distachyon* autotetraploid provided an additional polyploid to analyze the effects of chromosome duplication in *Brachypodium*.

### Differential gene expression between diploids and polyploids

**Figure 2.**
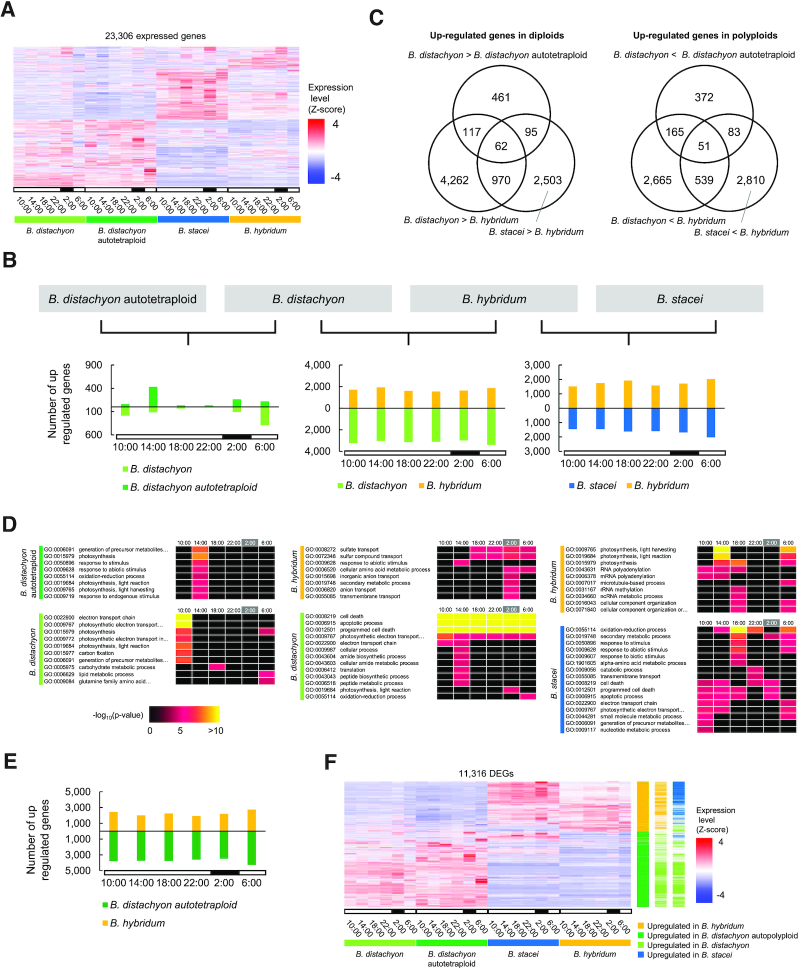
Comparative diurnal transcriptome analysis between diploid and polyploid *Brachypodium* species. (**A**) Hierarchically clustered heatmap of gene expression patterns of the diurnal transcriptome in *Brachypodium distachyon*, autotetraploid *B. distachyon*, *Brachypodium stacei* and *Brachypodium hybridum*. (**B**) Number of DEGs in diurnal transcriptomes between the *B. distachyon* diploid and autotetraploid, diploid *B. distachyon* and *B. hybridum* and *B. stacei* and *B. hybridum*. (**C**) Distribution of DEGs between diploids and polyploids. Venn diagrams represent the number of DEGs for each comparison. (**D**) GO terms for biological processes enriched in DEGs upregulated in each species. (**E**) Number of DEGs in diurnal transcriptomes between autotetraploid *B. distachyon* and *B. hybridum*. (**F**) Hierarchically clustered heatmap of gene expression patterns for DEGs identified between *B. distachyon* and *B. hybridum*. Color bars on the right side of the heatmap indicate species in which genes were upregulated.

To examine the transcriptional changes associated with polyploidization in *Brachypodium* species, we compared the diurnal transcriptomes of *B. distachyon*, *B. stacei*, *B. hybridum* and the artificially induced *B. distachyon* autotetraploid. We mapped RNA-seq reads from these four *Brachypodium* species to the Bd21 reference genome and to a virtual *B. stacei* genome generated by comparative analysis of homoeologous genomes in *B. hybridum*, *B. stacei* and *B. distachyon* (34) (Supplementary Table S1). We quantified gene expression on the basis of the annotated genes from the Bd21 genome. Overall, we detected 20 354, 20 876, 19 619 and 18 938 significantly expressed genes (RPM ≥ 1 in all biological replicates) in at least one time point in *B. distachyon*, *B. distachyon* autotetraploid, *B. stacei* and *B. hybridum*, respectively. We clustered gene expression patterns of these significantly expressed genes (a total of 23 306 genes, from the union of all expressed genes across *Brachipodium* species). The resulting hierarchically clustered heatmap of their Z-scored RPM-based gene expression patterns suggest high similarity in gene expression patterns between *B. distraction* and its autotetraploid, while exhibiting a clear difference between *B. hybridum* and its diploid progenitor species, *B. stacei* and *B. distachyon* (Figure 2A).

To assess gene expression differences between the diploids (*B. distachyon* and *B. stacei*) and polyploids (*B. distachyon* autotetraploid and *B. hybridum*), we performed pairwise comparisons of their diurnal transcriptomes at each time point (Figure 2B). We identified DEGs specifically upregulated in diploids or in polyploids (Figure 2C). As suggested by the heatmap of their transcriptome profiles, we detected many DEGs (FDR < 0.01) between *B. hybridum* and its diploid progenitor species, *B. distachyon* and *B. stacei*, at all time points: 8009 genes (34.4% of all expressed genes, *B. distachyon* > *B. hybridum* and/or *B. stacei* > *B. hybridum*) and 6313 genes (27.1% of expressed genes, *B. distachyon* < *B. hybridum* and/or *B. stacei* < *B. hybridum*) were upregulated in *B. distachyon* or *B. stacei* and upregulated in *B. hybridum*, respectively (Figure 2C). Comparing the diurnal transcriptomes of *B. distachyon* and the *B. distachyon* autotetraploid revealed 1416 DEGs (671 upregulated, 735 downregulated and 10 unclassified in *B. distachyon* autotetraploid), which is equivalent to 6.7% of all expressed genes in the corresponding transcriptomes. DEGs followed an uneven distribution across time points, especially those at 14:00 and 6:00 (Figure 2B), with more upregulated and downregulated genes in the *B. distachyon* autotetraploid relative to diploid *B. distachyon*, suggesting time-of-day specific gene expression changes in response to chromosome duplication.

We then explored the functions associated with DEGs between diploids and polyploids at each time point. As shown by a hypergeometric test-based GO enrichment analysis, enrichment in gene function for our DEGs showed overrepresentation of specific gene functions between diploids and polyploids at specific or all time points (Figure 2D). For example, photosynthesis-related functions such as photosynthetic light reaction and electron transport were enriched in all DEGs across different time points except for those upregulated in *B. hybridum* compared to *B. distachyon*. Indeed, we observed such overrepresentation of photosynthesis-related functions at 10:00 in *B. distachyon* and 14:00 in its autotetraploid, respectively. As reported in multiple comparative transcriptome studies between allopolyploids and diploids, stress response-related functions were enriched in genes upregulated in *B. hybridum* relative to *B. distachyon*, but also showed a time-of-day component. This pattern was more pronounced in *B. stacei* compared to *B. hybridum*, suggesting parental dependency. These findings illustrated functional differentiation between diploids and polyploids across their diurnal transcriptomes, suggesting their time-of-day specific representation.

Moreover, we compared the diurnal transcriptomes of the *B. distachyon* autotetraploid and the allopolyploid *B. hybridum*. We identified 11 316 DEGs composed of 6,667 upregulated and 4571 downregulated genes in the *B. distachyon* autotetraploid and 78 unclassified DEGs relative to *B. hybridum* (Figure 2E). We further compared the list of DEGs with those arising from comparisons between *B. hybridum* and its diploid progenitors, and discovered that most DEGs upregulated in the *B. distachyon* autotetraploid were also upregulated in diploid *B. distachyon* compared to *B. hybridum*, and those upregulated in *B. hybridum* were mainly upregulated in *B. stacei* (Figure 2F). These results also suggest that gene expression changes in the induced autotetraploid are small in response to chromosome duplication.

### HEB in the *B. hybridum* diurnal transcriptome

**Figure 3.**
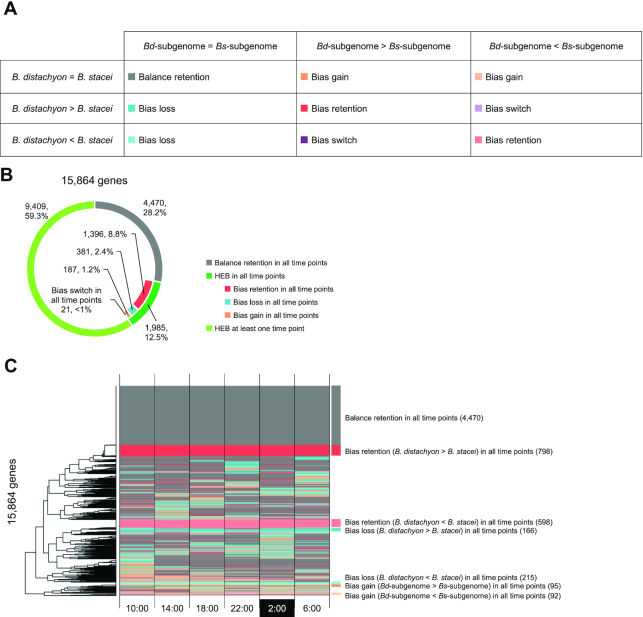
HEB in the *Brachypodium hybridum* diurnal transcriptome. (**A**) Categories of HEB pattern. (**B**) Number of genes classified into the categories of patterns of HEB events. In total, we classified 15 864 homoeologs expressed across all time points, based on the significance of differential expression with a false discovery rate set at ≤ 0.01 in DESeq2. (**C**) Hierarchically clustered patterns of HEB in the 15 864 homoeologs analyzed in (B). The patterns of HEB are colored by the categories shown in (A).

To investigate the HEB landscape in the diurnal transcriptome of *B. hybridum* compared to its diploid progenitors, we sorted RNA-seq reads into *Bd*- and *Bs*-subgenomes and compared the expression levels between homoeologous groups in the *B. hybridum* transcriptome. Using our paired-end RNA-seq datasets, we successfully assigned 81.3–87.9% of the RNA-seq reads to one or the other subgenome (Supplementary Figure S1), with 18 304 and 18 018 genes expressed in the *Bd*- and *Bs*-subgenomes, respectively. We assessed the patterns of HEB for 15 864 homoeologous groups that were expressed in at least one parental species and at least one subgenome in *B. hybridum* across all six time points. We classified the HEB patterns into five categories: balance retention, bias retention, bias gain, bias loss, and bias switch (Figure 3A and Supplementary Figure S2). We saw that 4470 (28.2%) gene groups were classified as balance retention (no HEB identified between diploid sister genomes and between subgenomes), while the remaining 11 394 (71.8%) gene groups showed HEB events between the diploid sister genomes and/or the subgenomes (Figure 3B). Of those, 1985 (12.5%) gene groups showed constitutive patterns of HEB events (8.8, 2.4, 1.2 and 0.1% of homoeologs classified as bias retention, bias loss, bias gain and bias switch categories, respectively) and 9409 (59.3%) gene groups showed HEB events for at least one time point. Hierarchical clustering of the HEB events illustrated highly temporal patterns of HEB events, which could be observed across all diurnal samples or at specific time points in the diurnal transcriptome (Figure 3C).

### HEB in diurnally expressed genes

**Figure 4.**
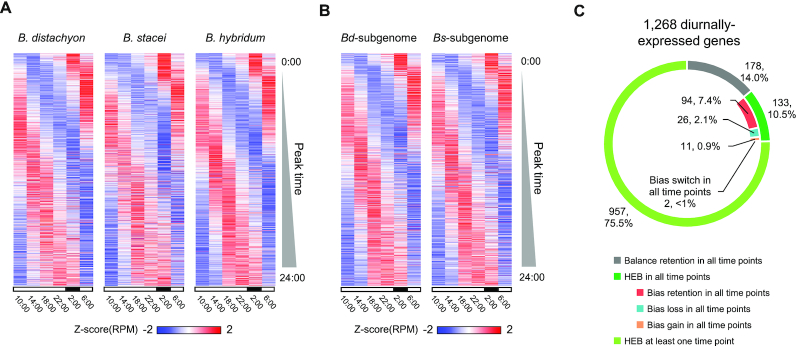
HEB of diurnally expressed genes. (**A** and**B**) Expression patterns of diurnally expressed genes identified in *Brachypodium distachyon* (1247 genes peak time estimated), *Brachypodium stacei* (1242 genes peak time estimated), *Brachypodium hybridum* (1258 genes peak time estimated) (A) and of their homoeolog counterparts in the *Bd*-subgenome (1253 genes peak time estimated) and *Bs*-subgenome (1255 genes peak time estimated). (B) The genes are sorted vertically as a function of their estimated peak time in each diurnal transcriptome dataset. (**C**) Number of genes classified into the categories of pattern of HEB events. In total, we classified 1268 diurnally expressed genes, based on the significance of differential expression with a false discovery rate set at ≤0.01 in DESeq2.

**Figure 5.**
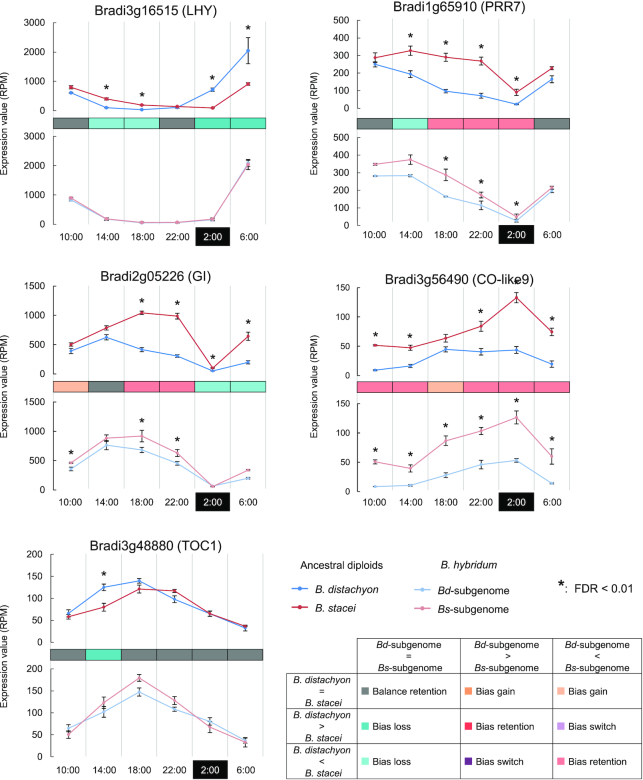
HEB of putative clock-related gene homologs and their counterparts in *Brachypodium stacei* and *Brachypodium distachyon*. Colored cells indicate patterns of HEB shown in Figure 3A. Expression patterns of homologs of *LHY* (At1g01060): Bradi3g16515, *GI* (At1g22770): Bradi2g05226, *TOC1* (At5g61380): Bradi3g48880, *PRR7* (At5g02810): Bradi1g65910, *CO-like9* (At3g07650): Bradi3g56490 are represented.

Since the analysis of conserved or altered HEB patterns in *B. hybridum* and its diploid progenitors illustrated a highly temporal component (Figure 3C), we further examined HEB in diurnally expressed genes. Through wavelet analysis of the diurnal transcriptome datasets from *B. distachyon*, *B. stacei* and *B. hybridum*, we identified 1268 diurnally expressed genes with a period of 24 h (*P* < 0.05 in all six permutations in at least one species). The diurnally expressed genes, sorted by their peak time of expression, covered the entire diurnal landscape (Figure 4A and Supplementary Table S2–4), which is a typical pattern for diurnally expressed genes. We confirmed that homoeologs of rhythmic genes in the *Bd*- and *Bs*-subgenomes of *B. hybridum* were equally rhythmic (Figure 4B; Supplementary Table S5 and 6). We then assessed HEB patterns for rhythmic homoeologs. In contrast to the HEB patterns observed in all expressed gene groups, diurnally expressed genes showed a higher proportion of HEB events (75.5% for at least one time point, and 10.5% at all time points). Moreover, we compared the expression levels of genes encoding putative clock-related gene homologs (based on the Arabidopsis annotation in TAIR10): *LATE ELONGATED HYPOCOTYL* (*LHY*, At1g01060, Bradi3g16515), *GIGANTEA* (*GI*, At1g22770, Bradi2g05226), *TIMING OF CAB2 EXPRESSION1* (*TOC1*, At5g61380, Bradi3g48880), *PSEUDO-RESPONSE REGULATOR7* (*PRR7*, AT5G02810, Bradi1g65910) and *CONSTANS-LIKE 9* (*CO-like9*, At3g07650, Bradi3g56490). These genes exhibited clear differences in their diurnal patterns between the diploid progenitors. However, expression patterns of homoeolog pairs were relatively similar in *B. hybridum*, resulting from bias loss at some time points, which may suggest the convergence of gene expression between homoeologs toward a similar diurnal phase (Figure 5).

**Figure 6.**
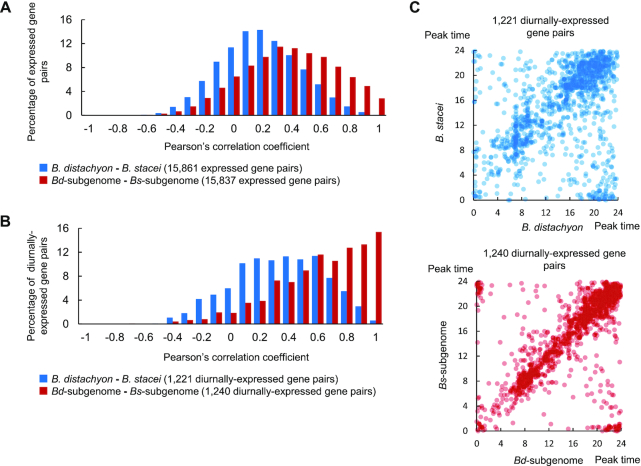
Comparative expression pattern of diurnally expressed genes. (**A** and**B**) Distribution of Pearson's correlation coefficients between *Brachypodium distachyon* and *Brachypodium stacei* and homoeologs in *Bd*-subgenome and *Bs*-subgenome. The distributions of Pearson's correlation coefficients were compared in the dataset composed of 15 861 and 15 837 expressed gene pairs in the diploid sister genomes and subgenomes, respectively, (A) and of 1221 and 1240 diurnally expressed gene pairs in the diploid sister genomes and subgenomes, respectively (B). (**C**) Scatterplots represent correlation of peak time of diurnally expressed genes between *B. distachyon* and *B. stacei*, and those homoeologs between the *Bd*-subgenome and *Bs*-subgenome.

To examine this convergence of harmonized phasing in gene expression between *B. hybridum* homoeologs in more detail, we compared the correlation in expression patterns of genes between the diploid progenitors, *B. distachyon* and *B. stacei*, and of homoeologs between the subgenomes in *B. hybridum*. We computed Pearson's correlation coefficients for expressed gene pairs (background gene set) and for diurnally expressed gene pairs. Both gene sets showed a shift in their distribution between the subgenomes to higher correlation coefficients when compared to their counterpart genes between the diploid progenitors, which suggests that gene expression patterns between the subgenomes in *B. hybridum* indeed may support their concerted changes in gene expression (Figure 6A and B). Interestingly, the shift in the distribution of correlation coefficients was more prominent in diurnally expressed genes (Figure 6B). To examine whether this similarity in expression patterns between diurnally expressed homoeologs in *B. hybridum* was due to averaging effects of both subgenomes or to dominance from one of the subgenomes, we also compared the distribution of correlation coefficients in the diurnally expressed genes between each of the *B. hybridum* subgenomes and each of the sister genomes of its diploid progenitors (Supplementary Figure S3). We observed no biased distribution of correlation coefficients across orthologous comparisons: *B. distachyon*—*Bd*-subgemome and *B. stacei*—*Bs*-subgenome (Supplementary Figure S3A), as well as the reciprocal comparisons, *B. distachyon*—*Bs*-subgemome and *B. stacei*—*Bd*-subgenome (Supplementary Figure S3A), suggesting that the expression similarity between diurnally expressed homoeologs in *B. hybridum* largely results from averaging effects between the expression levels of homoeologous genes. Moreover, we compared the distribution of peak expression time estimated for the diurnally expressed genes in both the diploid sister genomes and each subgenome. Their peak time was more highly correlated between the subgenomes than between the diploid sister genomes (Figure 6C). These findings suggest that the harmonized diurnal phasing of gene expression between homoeologs may be widespread in *B. hybridum*, and may result in their coordinated participation in gene regulatory networks across subgenomes.

## DISCUSSION

### Gene expression divergence in diurnal transcriptomes across *Brachypodium* cytotypes

In this study, we analyzed the diurnal transcriptomes of the natural allopolyploid *B. hybridum* and its diploid progenitor species, *B. distachyon* and *B. stacei*, as well as an artificially induced *B. distachyon* autotetraploid. Our comparative transcriptome analysis between diploid and autotetraploid *B. distachyon* identified a small number of DEGs in the diurnal transcriptome datasets (∼6.1% of the expressed genes, Figure 2B and C). Because the autotetraploid *B. distachyon* Bd21 was induced by colchicine treatment of a diploid Bd21, its duplicated genes have accumulated no polymorphisms and are identical in sequence to the diploid parent Bd21 (Figure 1C). This feature of our induced autotetraploid allowed us to estimate the dosage effect across the diurnal transcriptome of duplicated genes due to chromosome doubling. Our results support the hypothesis that autopolyploids carry fewer genomic alterations than allopolyploids due to the absence of hybridization, which was proposed as a major source of polymorphisms in polyploids compared to autopolyploids (27). However, the magnitude of gene expression changes also depends significantly on the diploid parents (39), developmental stages and organs (22) and environmental conditions (40). Our comparative transcriptome analysis between diploid and autotetraploid *B. distachyon* during a diurnal time course demonstrated that the magnitude of gene expression changes also depends on sampling time (Figure 2B), suggesting the presence of transcriptional perturbations with rhythmic (diurnal and/or circadian) and/or time-specific expression. We observed phenotypic differences in seeds between the diploid and autotetraploid *B. distachyon* Bd21 (Figure 1E and D). Further intraspecific comparative studies between diploids and autopolyploids will be required to determine the effects of WGD on gene expression and their relation to morphological and physiological changes that may occur under particular growth stages, organs and environmental conditions. In contrast to the less severe transcriptional alterations observed between diploid *B. distachyon* and its autotetraploid, we identified many DEGs across the diurnal cycle between *B. hybridum* and its diploid progenitors (Figure 2B), in agreement with our previous transcriptome studies in leaves and roots across these cytotypes (34). Most DEGs were specific for each comparison between *B. hybridum* and the diploid progenitors, *B. distachyon* and *B. stacei* (Figure 2A and C), which suggests that severe transcriptome divergence occurred among the subgenomes of *B. hybridum* and diploid sister genomes of *B. distachyon* and *B. stacei* over the course of their independent evolutionary trajectories. Therefore, in addition to the dosage effect of duplicated genes, the altered gene expression patterns observed between *B. hybridum* and its diploid progenitors may be related to the divergence of their ancestral genomes, and thus of their transcriptomes, since the separation of the *B. stacei* lineage (∼10 Mya) and the *B. distachyon* lineage (∼7 Mya). In addition, each subgenome in *B. hybridum* may have undergone further divergence since the allopolyploidization of *B. distachyon* and *B. stacei* (∼1 Mya). A final potential source of variation between subgenomes may also have resulted from consequences of allopolyploidy such as the combined effects of *trans*- and *cis*-acting factors from both subgenomes (28). Although we cannot dissect the relative contribution of each of these possible effects on genome and transcriptome divergence between diploids and their historically derived allopolyploid lineage, further comparative genome and transcriptome studies with these cytotypes and with artificially induced *B. hybridum* by crosses between *B. distachyon* and *B. stacei* (50) may provide insight into the long-term and immediate effects of allopolyploidization.

### Retained and altered HEB in the *B. hybridum* diurnal transcriptome

Our homoeolog-specific gene expression analysis demonstrated the complexity of parental legacy and hybrid novelty in the *B. hybridum* transcriptome. HEB is a widely observed transcriptional phenomenon in allopolyploids, and its conserved pattern between allopolyploid subgenomes and diploid sister genomes has been examined in the context of parental legacy and gene expression novelty in allopolyploid transcriptomes (26,42). In this study, we determined that 8.8% of the 15 864 homoeologous gene groups expressed in the *Brachypodium* cytotypes showed conserved HEB between the *B. hybridum* subgenomes and between the diploid sister genomes of *B. distachyon* and *B. stacei* (bias retention) throughout all six diurnal time points (Figure 3B and C). These results suggest that parental legacies of gene expression are still detectable across the lineage of the *Brachypodium* cytotypes, even ∼1 Mya after their allopolyploidization. Previous work across various allopolyploids has shown that HEB varies between tissues (52), developmental stages (43,44) and environmental conditions (45). We also previously reported on HEB in *B. hybridum* leaves and roots, as well as in leaves subjected to heat stress (28). In the present study, we identified a time-dependent pattern of HEB, which may result from phase and/or amplitude differences between diurnally expressed homoeologs (Figure 3B and C). Assessing HEB in rhythmically expressed genes, we observed that as much as 86% of rhythmic genes displayed HEB in at least one time point or at all time points (Figure 4C). Time-dependent HEB, observed in putative homologs of the Arabidopsis clock-related genes *LHY*, *TOC1*, *GI*, *PRR7* and *CO-like9*, demonstrated clear differences in diurnal patterns (Figure 5), which might be a consequence of adaptation to different habitats. Moreover, we observed that expression patterns in homoeologs of these clock-related genes in *B. hybridum* were relatively similar. These genes likely encode components of the plant circadian clock (46) or are regulated by the circadian clock (53), which comprise multiple transcriptional feedback loops and may therefore cause expression similarity between diurnally expressed homoeologs in the *B. hybridum* transcriptome by balancing the expression levels and harmonizing the diurnal phases across homoeologs.

### Expression similarity between diurnally expressed homoeolog Pairs in *B. hybridum*

In the present study, we demonstrated that the time of expression-peak (or phase) of diurnally expressed homoeologs was synchronized in *B. hybridum*, although they were different between their progenitors, which may strengthen the correlation of their expression patterns (Figure 6). This finding is consistent with stabilizing selection, in which gene expression divergence by *cis*-regulatory divergence in progenitors can be reduced by *cis-trans* compensatory effects in hybrids or allopolyploids (54). After two diploid progenitors speciate and undergo independent evolutionary trajectories (possibly including diverging circadian regulation of gene expression through adaptation to ecological niches across latitude (55)), their hybridization would bring together distinct *trans*-acting regulatory factors involved in circadian regulation from both parental subgenomes. This set of *trans*-acting factors will likely regulate various downstream homoeologs on both subgenomes and compensate for parental *cis*-regulatory divergence by averaging out subgenome-specific *trans* effects, resulting in expression convergence between diurnally expressed homoeolog pairs in the allopolyploid. A study on *cis*- and *trans*-regulation in Arabidopsis allopolyploids demonstrated that compensatory *cis*-*trans* effects were enriched in genes associated with biosynthetic and metabolic processes (56). Studies on robustness of transcriptional regulation in yeast (*Saccharomyces cerevisiae*) and the fruit fly (*Drosophila melanogaster*) have suggested that *cis*-regulatory mutations can be compensated by *trans*-regulatory feedback mechanisms, facilitating mutational robustness (57, 58). These studies and our data may suggest that gene expression regulation by a *trans*-regulated feedback mechanism can be stabilized through *cis-trans* compensatory effects in allopolyploids. In Shepherd's purse (*Capsella bursa-pastoris*), an allopolyploid weed, a comparative transcriptome analysis between its subgenomes showed not only balanced homoeolog expression between its subgenomes but also ELD that varied across tissues (59). Although our data did not suggest subgenome dominance in the expression similarity between diurnally expressed homoeolog pairs in *B. hybridum*, further work across various tissues and environmental conditions may illustrate different modes of regulatory novelties in *B. hybridum*. Here, we analyzed transcriptome datasets from diurnal samples collected every 4 h from plants grown under long-day conditions (20 h light/4 h dark diurnal cycles). Some of the differences observed in the transcriptomes of the diploid progenitors might therefore reflect their diverging daylength responsiveness. Naturally diversified daily rhythms, especially in the context of phase differences in gene expression, can modify traits of ecological importance (47). Indeed, hybridization of two species with different daily rhythms may cause novel (averaging) rhythmicity, which will influence a wide range of physiological functions and lead to potential phenotypic innovation for allopolyploids.

## DATA AVAILABILITY

The RNA-seq data are archived at the DNA Data Bank of Japan under accession number DRA008910. Whole-genome sequencing data for the *B. distachyon* Bd21 autotetraploid are archived at the DNA Data Bank of Japan under accession number DRA008925. The datasets and script codes used in this study are freely available at https://github.com/BioproductivityInformaticsResearchTeam/Inoue_and_Takahagi_etal_NARGAB-2020/.

## Supplementary Material

lqaa067_Supplemental_File
