# Supplementary material for: Parental legacy and regulatory novelty in Brachypodium diurnal transcriptomes accompanying their polyploidy
Source: NAR Genom Bioinform. 2020 Sep 22;2(3):lqaa067. doi: 10.1093/nargab/lqaa067 (PMC7671347; doi:10.1093/nargab/lqaa067)
Supplement: lqaa067_Supplemental_File [file lqaa067_supplemental_file.pdf]

SUPPLEMENTARY FIGURES

Parental Legacy and Regulatory Novelty in *Brachypodium* Diurnal Transcriptomes Accompanying their Polyploidy

Komaki Inoue[1]†, Kotaro Takahagi[1,2,3]†, Yusuke Kouzai[1], Satoru Koda[4], Minami Shimizu[1], Yukiko Uehara-Yamaguchi[1], Risa Nakayama[1], Toshie Kita[1], Yoshihiko Onda[1], Toshihisa Nomura[1,5], Hidetoshi Matsui[6], Kiyotaka Nagaki[7], Ryuei Nishii[8] and Keiichi Mochida[1,2,3,5,7,*]

1 RIKEN Center for Sustainable Resource Science, Tsurumi-ku, Yokohama, Japan
2 Kihara Institute for Biological Research, Yokohama City University, Totsuka-ku, Yokohama, Japan
3 Graduate School of Nanobioscience, Yokohama City University, Tsurumi-ku, Yokohama, Japan
4 Graduate School of Mathematics, Kyushu University, Fukuoka, Japan
5 RIKEN Baton Zone Program, Tsurumi-ku, Yokohama, Japan
6 Faculty of Data Science, Shiga University, Hikone, Japan
7 Institute of Plant Science and Resources, Okayama University, Kurashiki, Japan
8 School of Information and Data Science, Nagasaki University, Nagasaki, Japan

*Corresponding Author: Tel: +81 (0)45 503 9111; Fax: +81 (0)45 503 9609; Email: keiichi.mochida@riken.jp
† Joint first authors

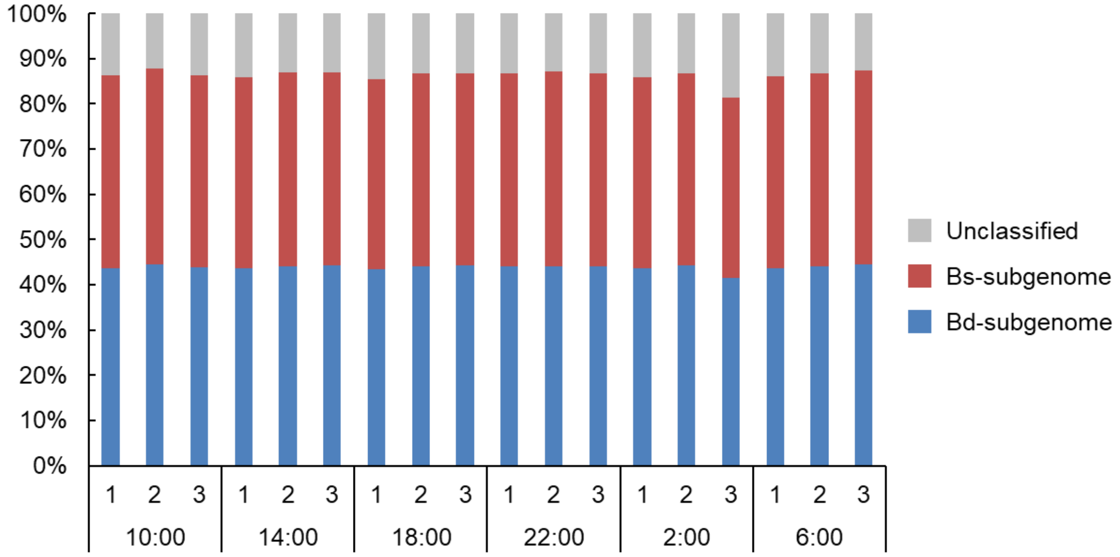

Supplementary Figure S1: RNA-seq read assignment to *B. hybridum* subgenomes for each time point in the diurnal transcriptome. The bars show the percentage of the *Bd*-subgenome reads (blue), *Bs*-subgenome reads (red), and unclassified reads (grey) of the *B. hybridum* RNA reads.

HEB category	*B. distachyon* – *B. stacei*	*Bd*-subgenome – *Bs*-subgenome	Relative expression patterns
Balance retention	*B. distachyon* = *B. stacei*	*Bd*-subgenome = *Bs*-subgenome	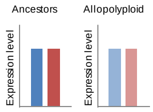
Bias gain	*B. distachyon* = *B. stacei*	*Bd*-subgenome > *Bs*-subgenome	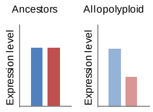
Bias gain	*B. distachyon* = *B. stacei*	*Bd*-subgenome < *Bs*-subgenome	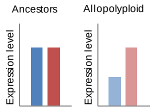
Bias loss	*B. distachyon* > *B. stacei*	*Bd*-subgenome = *Bs*-subgenome	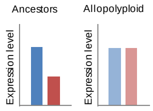
Bias retention	*B. distachyon* > *B. stacei*	*Bd*-subgenome > *Bs*-subgenome	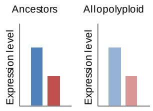
Bias switch	*B. distachyon* > *B. stacei*	*Bd*-subgenome < *Bs*-subgenome	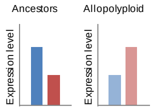
Bias loss	*B. distachyon* < *B. stacei*	*Bd*-subgenome = *Bs*-subgenome	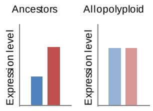
Bias switch	*B. distachyon* < *B. stacei*	*Bd*-subgenome > *Bs*-subgenome	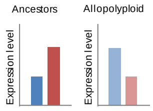
Bias retention	*B. distachyon* < *B. stacei*	*Bd*-subgenome < *Bs*-subgenome	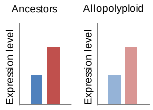

B. distachyon
B. stacei

Bd-subgenome
Bs-subgenome

Supplementary Figure 2: Expected expression patterns in each HEB category. The HEB patterns are colored according to the categories shown in Figure 3A.

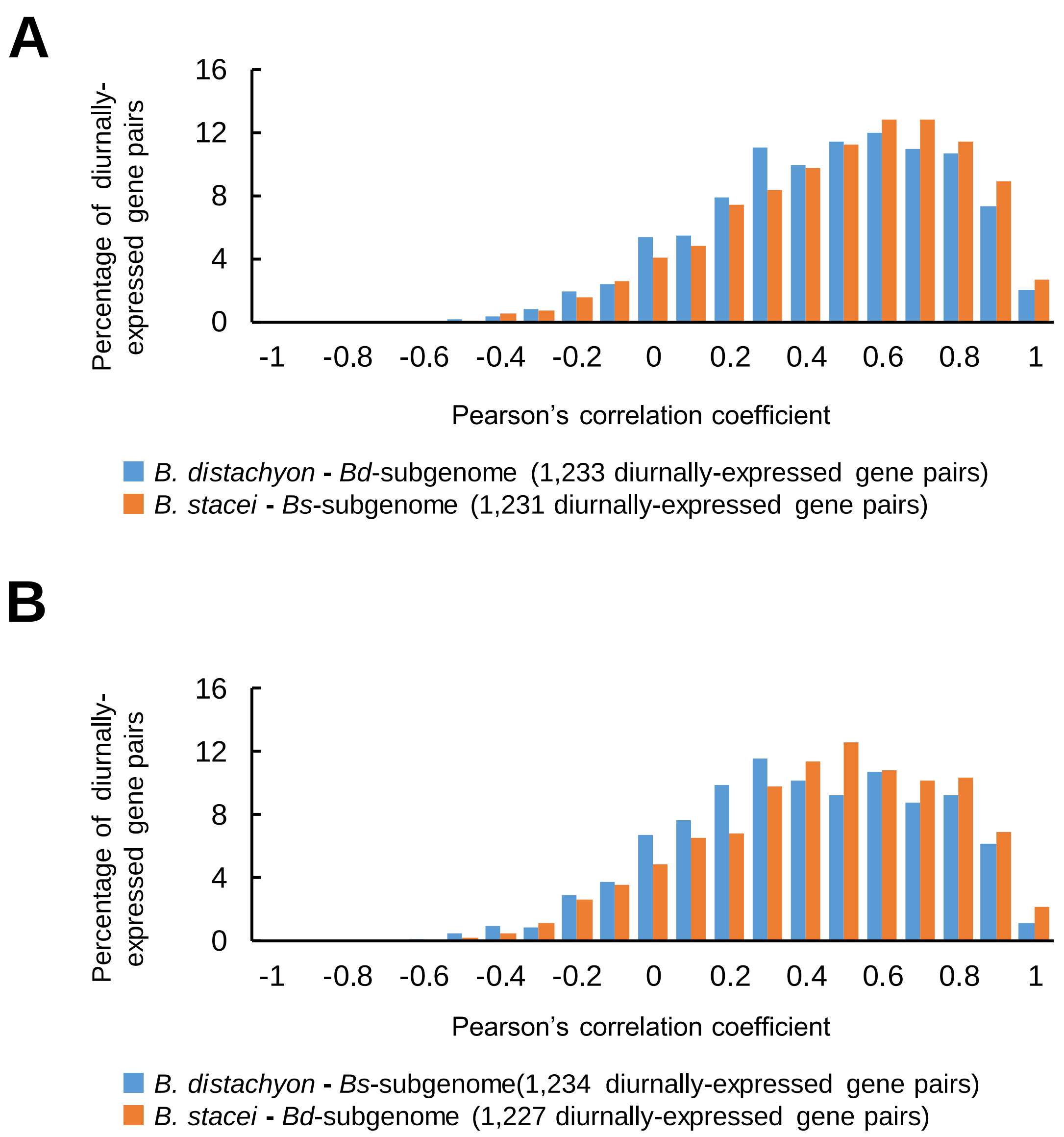

Supplementary Figure 3: Distribution of Pearson's correlation coefficients in diurnally-expressed homoeologues between each of the *B. hybridum* subgenomes and the sister genomes of its diploid progenitors. (**A**) Orthologous comparison of 1,233 and 1,231 diurnally-expressed gene pairs in *B. distachyon* – *Bd*-subgemome and *B. stacei* – *Bs*-subgenome, respectively. (**B**) Reciprocal comparison of 1,234 and 1,227 diurnally-expressed gene pairs in *B. distachyon* – *Bs*-subgemome and *B. stacei* – *Bd*-subgenome, respectively.
